# Iran’s success in controlling the COVID-19 pandemic

**DOI:** 10.1017/ice.2020.169

**Published:** 2020-04-23

**Authors:** Nima Mohammadzadeh, Mahla Shahriary, Erfan Nasri

**Affiliations:** 1 Department of Microbiology, Faculty of Science, Shahid Beheshti University, Tehran, Iran; 2 Student Research Committee, Iran University of Medical Sciences, Tehran, Iran; 3 Department of Microbiology, Tarbiat Modares University of Medical Sciences, Tehran, Iran; 4 Student Research Committee, School of Medicine, Guilan University of Medical Sciences, Rasht, Iran

**Fig. 1.**
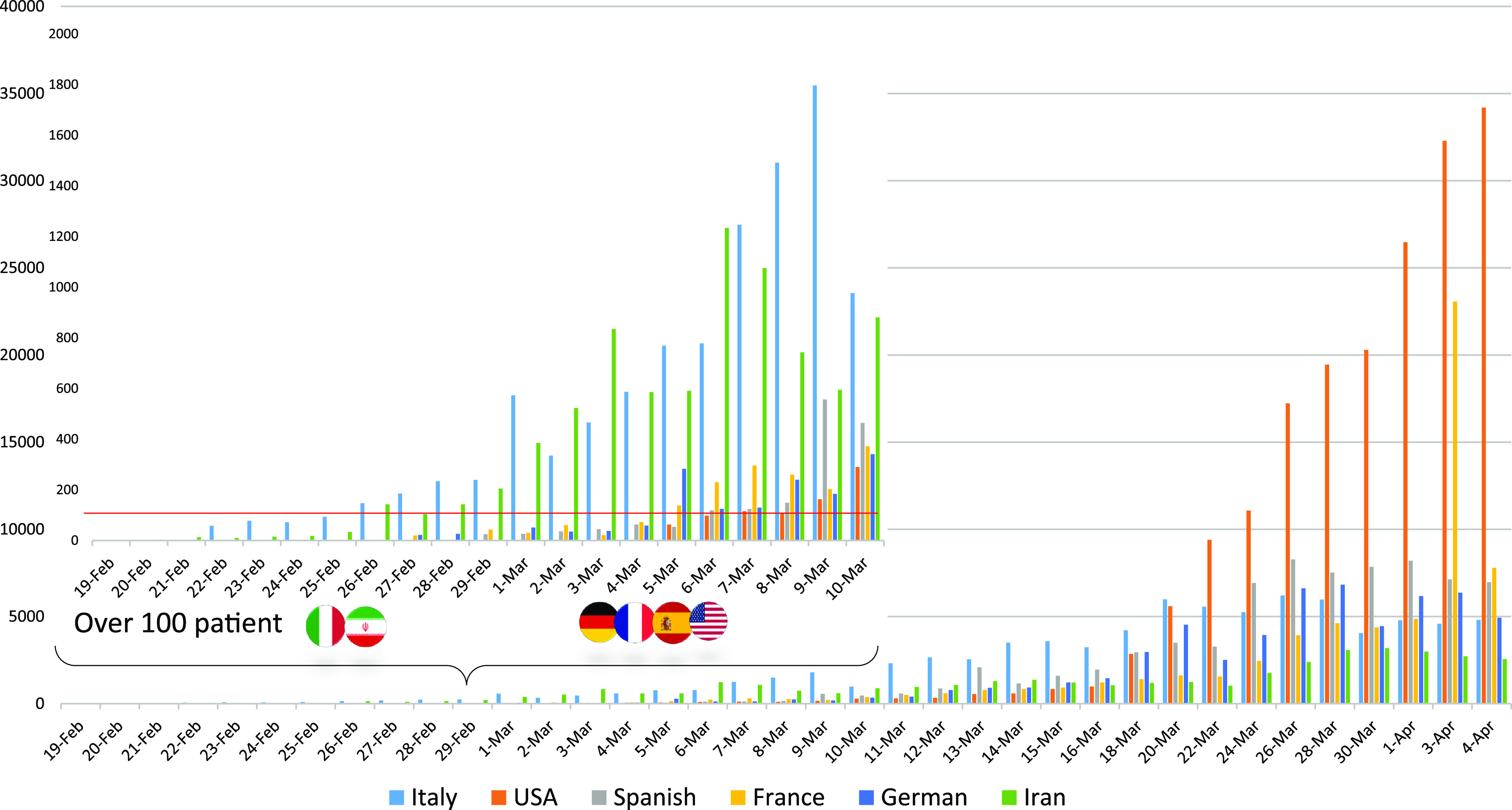
Daily New Cases.

*To the Editor*—Coronavirus disease 2019 (COVID-19) is a respiratory tract infection ranging from mild respiratory illness (eg, respiratory symptoms, cough, fever, shortness of breath and breathing difficulties) to severe illness (eg, pneumonia, severe acute respiratory syndrome, kidney failure, and death)^1^ that has caused an unprecedented global crises in <90 days in all 206 countries of the world.^2^ Today, most of the world’s major cities are in full quarantine and all social and economic behaviors have been limited due to the SARS-Cov-2 outbreak. Controlling the spread of the virus has become one of the most important challenges for governments across the globe. The increase in COVID-19 cases in the advanced industrial countries, including Italy, Germany, France, Spain, and United States, reflects the rapid spread of the virus. As of April 4, 2020, the following countries have been most affected: Italy, with a populations of almost 60 million, has ~119,827 COVID-19 patients (case fatality rate [CFR], 12.25%). Germany, with a population of almost 82 million, has ~85,778 COVID-19 patients (CFR, 1.34%). France, with a population of almost 66 million, has ~63,536 COVID-19 patients (CFR, 10.21%). Spain, with a population of almost 46 million, has ~117,710 patients (CFR, 9.28%). And the United States, with a population of 320 million, has ~241,703 patients (CFR, 2.42%). The prevalence of SARS-CoV-2 is rapidly increasing. Iran, with a population of almost 81 million, has ~44,605 COVID-19 patients, with 2,898 deaths (Fig. 1). Although Iran has been heavily sanctioned in all fields of industry and pharmacy, it has taken important steps from the earliest days of the outbreak to combat the virus.^3^

For example, Italy, with an outbreak date similar to that of Iran, has more than twice the disease incidence rate of Iran. According to reports released by the ministries of health in Iran and Italy,^4,5^ Iran formed a headquarters for the COVID-19 crisis on February 23, when the virus count was ~15 people per day. In contrast, in Italy, the COVID-19 crisis headquarters was formed on March 13, when the outbreak count was ~2,500 per day. Also, these countries’ respective health ministries published safety and prevention guidelines for many locations, especially crowded centers including hospitals, clubs, transportation systems, schools, etc, in the early days of the outbreak. They also sought widespread collaboration with NGOs and volunteers as well as extensive intragovernmental collaboration to ensure the observation of safety protocols to control the spread of disease. Although traffic and concentration laws as well as heavy fines were not considered in the early days, these collaborations ultimately resulted in an 80% reduction in traffic between cities and as well as in social gatherings and even family gatherings. Ultimately, all of these measures have led Iran to better control the spread of the virus than other aforementioned industrialized countries. Nevertheless, Iran has a long way to go to achieve complete control of the pandemic.

Because Iran is located among neighboring countries in a very high-risk area for many diseases, including tuberculosis, rabies, Crimean Congo fever, cholera, brucella, malaria, polio, and some others, it has been even more successful in controlling such diseases than the United States.^6^ This experience and history are expected to be very useful and effective in controlling COVID-19.
